# Machine-learning-driven rational design of gelatin architectures: saccharide derivatives as structural plasticizers under low water content

**DOI:** 10.1039/d6ra06070f

**Published:** 2026-09-15

**Authors:** Gaku Yazawa, Masato Masuda, Shigehito Osawa

**Affiliations:** a Department of Biomedical Engineering, Graduate School of Life Science, Toyo University 48-1 Oka, Asaka Saitama 351-8510 Japan osawa@toyo.jp; b Department of Information Science and Arts, Faculty of Information Science and Arts, Toyo University 2100 Kujirai, Kawagoe Saitama 350-8585 Japan masuda061@toyo.jp; c Department of Biomedical Engineering, Faculty of Life Science, Toyo University 48-1 Oka, Asaka Saitama 351-8510 Japan

## Abstract

Saccharide derivatives are highly promising, sustainable additives for tuning the physical profiles of gelatin-based green biomaterials. Although they are utilized in several applications in medical and food sciences, their structural roles under highly dehydrated conditions remain poorly understood. Here, we investigated the influence of various saccharide derivatives on gelatin networks across a broad structural spectrum, from bulk hydrogels to low-moisture sheets. Rheological measurements of the hydrogels revealed that the addition of saccharide derivatives elevated the gelation temperature and facilitated the network formation by restricting free water through preferential hydration. Intriguingly, this behavior inverted in the dried sheet state, where ATR-FTIR spectroscopy revealed that the saccharides disrupted the gelatin network to function as molecular plasticizers, significantly enhancing flexibility and extensibility. While residual water retained by saccharide hydration mainly drives this plasticization as previous study predicted, water amount alone fails to account for notable mechanical variations; for example, sorbitol-doped sheets required significantly less residual water to achieve equivalent elasticity compared to sucrose systems. To address this complexity, we leveraged a machine-learning-based Random Forest Regression (RFR) model. The RFR successfully decoupled the overlapping effects of water content and molecular features, identifying molecular volume and hydrogen-bond donor of the additives as the governing factors of mechanical properties. This predictive, data-driven framework offers new insights for the rational design of eco-friendly gelatin biomaterials with precisely tailored mechanics.

## Abstract 2

Saccharide derivatives improve extensivity and flexibility of sheets fabricated from gelatin hydrogels, where key factors are determined by machine learning.

## Graphical abstract

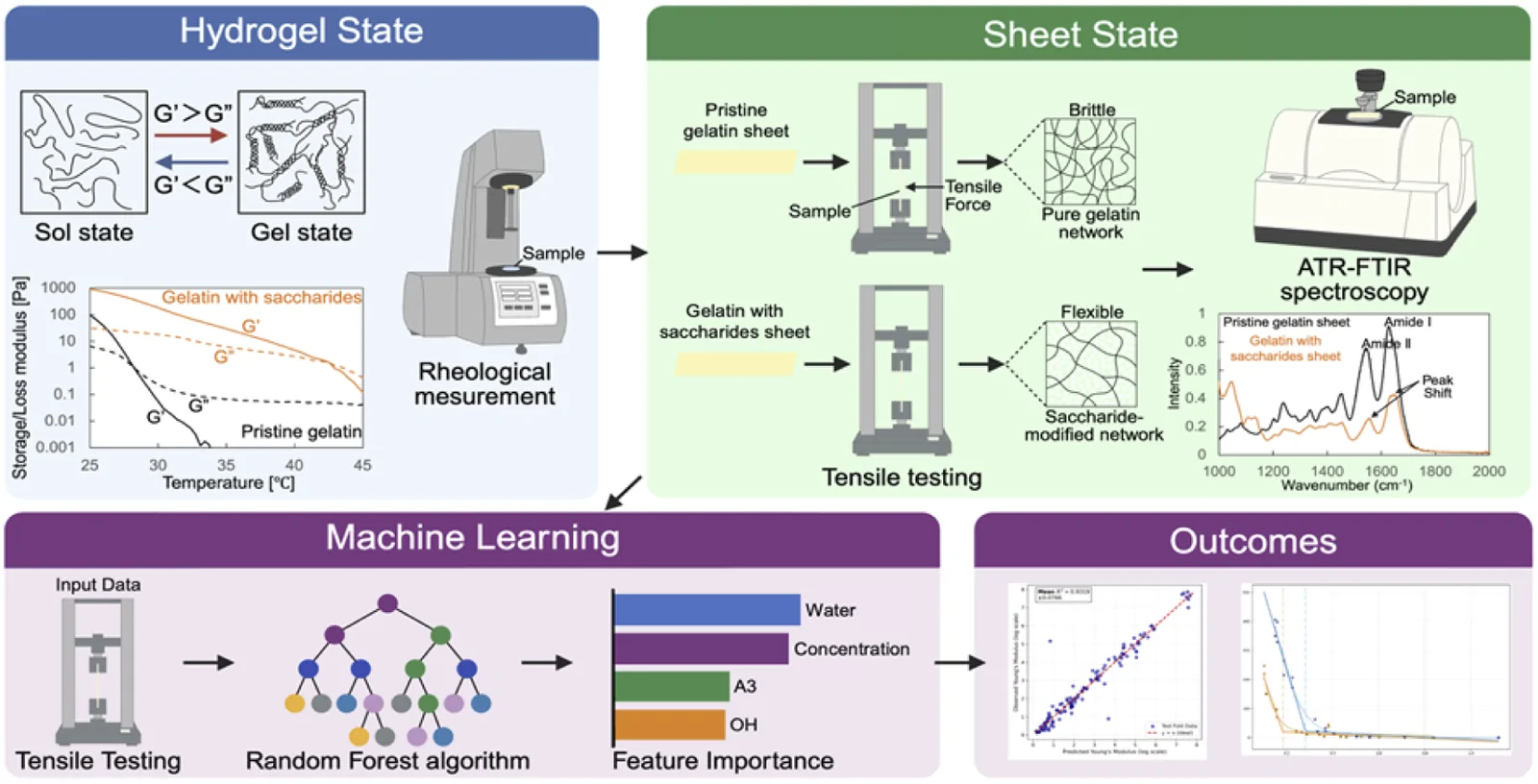

## 1. Introduction

Gelatin, a natural biopolymer derived from collagen, has been widely utilized in various fields of applied chemistry, ranging from biomaterials and drug delivery systems to soft robotics and edible sheets.^1–6^ In these practical applications, modulating their viscoelastic and mechanical properties has been one of the main topics of research because gelatin-based materials generally lack mechanical strength and are often fragile.^7–9^ Although several approaches have been developed such as introducing chemical cross-linking, inducing chemical modification of gelatin, and building other polymer networks,^7–17^ simply adding nature-derived products to control these parameters is more favorable from the perspective of green chemistry.

Phenomenologically, the addition of saccharides to gelatin solutions is known to elevate the gelation temperature and increase the stiffness of the resulting networks.^18–20^ Recent advanced physicochemical studies have elucidated the underlying molecular mechanism; saccharide molecules exhibit strong preferential hydration, effectively sequestering water molecules away from the gelatin chains.^20^ This water deprivation causes a micro-phase separation or a localized crowding of the gelatin, meaning that the gelatin network behaves as if it were at a much higher concentration than its nominal formulation. This fundamental mechanism directly correlates with the macroscopic behavior observed during the dehydration of gelatin hydrogels.

When water is systematically removed from a hydrogel—which inherently increases the actual concentration of the gelatin—the mechanical stiffness increases as a natural consequence of denser network formation, ultimately resulting in the transition of the gel to a sheet.^12,21–23^ This extremely low-water condition of the sheets does not feature a typical hydrated hydrogel network but rather a highly concentrated and water-deficient state of the gelatin complex. In such restricted environments, pristine gelatin (PG) forms stiff but fragile sheets. Importantly, the addition of saccharides alters the mechanical properties of gelatin sheets, rendering them more extensible by inducing a plasticization effect. In this state, the saccharide molecules are considered to modulate the mobility of gelatin chains within the sheet, either by binding an appropriate amount of water or by acting directly as plasticizers themselves.^18–20^ However, it remains an open and critical question whether specific control factors emerge to govern the mechanical properties of the sheets under these extremely low-water conditions. Crucially, the macroscopic flexibility of these sheets is governed not only by a slight increase in residual water content retained by the added saccharides but is also modulated by the specific molecular structures of the co-existing saccharides.

Understanding and unravelling these intricate phenomena under low-water conditions is not merely an academic exercise; it holds profound implications for green chemistry. Deciphering the precise roles of solvent water and saccharides would lead to next-generation, sustainable, and entirely nature-derived materials without relying on synthetic crosslinkers or hazardous organic solvents. Thus, this study systematically investigates the joint effects of water content and various saccharide derivatives—which exhibit diverse effects on gelatin hydrogel properties—on the mechanical characteristics of the resulting gelatin sheets. To untangle these multi-dimensional, non-linear interactions, data-driven approaches leveraging machine learning offer significant advantages over traditional statistical methods. Conventional analyses often fail to isolate individual contributions when factors such as residual water content and chemical structure are strongly coupled. Here, we employed Random Forest Regression (RFR), an ensemble machine learning technique,^24–26^ which is particularly suited for capturing complex, non-linear relationships without assuming predefined functional forms. Furthermore, RFR enables robust quantitative ranking of feature importances (*e.g.*, *via* mean decrease in impurity and permutation importance), thereby providing a clear predictive and mechanistic basis to decouple the overlapping effects of macroscopic processing variables and microscopic molecular features (Fig. 1).

**Fig. 1:**
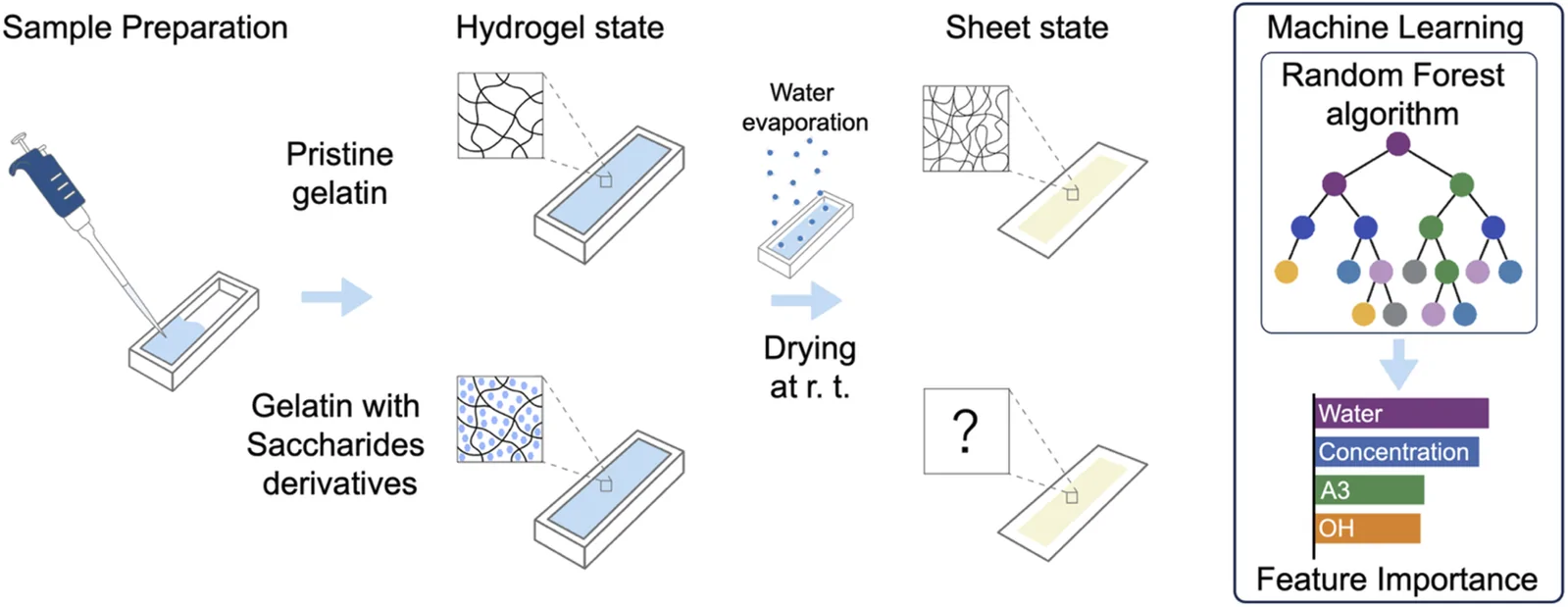
Schematic illustration of material preparation of this study.

## 2. Materials and methods

### 2.1. Materials

Type A gelatin derived from porcine skin was obtained from Nitta Gelatin Inc. (Osaka, Japan). Sucrose, d(+)-glucose, and d(–)-sorbitol were purchased from FUJISHEET Wako Pure Chemical Corporation (Osaka, Japan). d(–)-Fructose was purchased from Tokyo Chemical Industry Co., Ltd. (Tokyo, Japan). All chemicals were used as received without further purification.

### 2.2. Preparation of gelatin hydrogels

A 20 wt% gelatin solution was first prepared in a beaker and allowed to swell at room temperature for 30 min. After swelling, the gelatin solution was heated in a water bath at 50 °C for 30 min until completely being dissolved. Saccharides and sorbitol solutions were separately prepared in screw-capped bottles at concentrations of 20, 40, and 60 wt%. These solutions were dissolved using a hot stirrer at 60 °C and 500 rpm for 30 min. Subsequently, the 20 wt% gelatin solution and each saccharide or sorbitol solution were mixed at a volume ratio of 1 : 1 (v/v) in screw-capped bottles. The final hydrogel compositions were adjusted to contain 10 wt% gelatin and 10, 20, or 30 wt% saccharides and sorbitol, respectively.

### 2.3. Rheological evaluation of the gelatin hydrogels

The gel precursor samples were heated at 50 °C for 10 min and mixed using a vortex mixer for 30 s. Three mL of the samples were then placed on the rheometer (MCR302e, Anton Paar, Graz, Austria) maintained at 45 °C and equilibrated for 5 min to ensure temperature uniformity. Then, oscillatory measurements were performed using a cone–plate geometry (diameter: 50 mm, cone angle: 1°) with a gap distance of 1 mm. The oscillatory measurements were conducted at a frequency of 1 Hz and a strain of 1%, while the temperature was decreased from 45 °C to 25 °C at a cooling rate of 0.5 °C min^−1^. The gelation temperatures were defined as the crossover point of the storage modulus (*G*′) and loss modulus (*G*″), indicating the transition between sol and gel states.

### 2.4. Preparation of gelatin sheets

The gelatin solutions were prepared as described the above to obtain hydrogels containing 10 wt% gelatin and 10, 20, or 30 wt% saccharides or sorbitol. Subsequently, 5 g of each prepared hydrogel was cast into a rectangle mold with dimensions of 20 mm × 80 mm and incubated at room temperature (25 °C, ∼40% humidity) for 24 h to form gelatin sheets.

### 2.5. Tensile strength test of the gelatin sheets

The tensile properties of the gelatin sheets were evaluated according to ASTM D882 standard. The gelatin sheets were cut into specimens measuring 15 mm × 70 mm. The thickness of each specimen was measured at four different positions using a digital calliper, and the average value was used as the specimen thickness. Tensile tests were performed using a universal testing machine (MCT-2150W, A&D Co., Ltd., Tokyo, Japan) equipped with a 500 N load cell. The initial grip separation was set to 50 mm, and the crosshead speed was fixed at 60 mm min^−1^. Young's modulus was determined from the stress–strain curves obtained during the tensile tests.

### 2.6. Fourier transform infrared (FTIR) spectroscopy analysis of the gelatin sheets

Fourier transform infrared (FTIR) spectroscopy was performed to analyze the chemical structure of the gelatin sheets. The gelatin sheets prepared as described above were analyzed using an FTIR spectrometer (Nicolet iS5, Thermo Fisher Scientific, Waltham, MA, USA) equipped with an iD7 ATR Diamond accessory. Prior to sample measurements, a background spectrum was collected and used for automatic background correction of all subsequent spectra. Spectra were collected in the range of 4000–400 cm^−1^ with a resolution of 1 cm^−1^ and 32 scans per sample. Peak assignments and spectral interpretations were performed based on the report by Shurvell *et al.*^27^

### 2.7. Machine learning modeling and descriptor generation

A total of 122 experimental data points were compiled from the library of gelatin–saccharide composite sheets (Tables S1–S3). Sample names are defined by amount and species of saccharide derivatives (Suc: sucrose, Glu: glucose, Fru: fructose, Sor: sorbitol). For example, Suc10 is the name of sheet prepared from solutions containing gelatin 10 wt% and sucrose 10 wt%. PG is the sheet prepared without adding saccharide derivatives. The dataset encompasses various formulation conditions: pure gelatin (*n* = 7), and gelatin with four types of saccharide derivatives (sucrose, glucose, fructose, and sorbitol) at three distinct target concentrations (10, 20, and 30 wt% relative to gelatin; *n* ≈ 10 or 9 for each condition), each yielding localized variations in residual water content. To quantitatively predict the Young's modulus of sugar-doped gelatin sheets, a machine learning regressor based on the Random Forest Regressor (RFR) algorithm was implemented using the scikit-learn library in Python.^24^ To maintain model simplicity and eliminate arbitrary tuning bias, standard default hyperparameters were employed (including unconstrained tree depth and default node-splitting criteria). The number of trees (*n*_estimators_) was intentionally set to 1000 to maximize ensemble stability and minimize prediction variance during cross-validation. Notably, despite using these un-tuned baseline parameters, the model achieved a high coefficient of determination (*R*^2^), demonstrating that the selected chemical descriptors natively capture the essential features governing the mechanical properties without requiring intensive algorithmic optimization. Experimental data obtained from five distinct sugar types—sucrose (suc), glucose (glu), fructose (fru), sorbitol (sor), and a sugar-free control (pg)—under various processing times were compiled. To represent the chemical and structural characteristics of the additive matrix, each sugar type was encoded using seven molecular descriptors computed *via* the RDKit^25^ library: molecular weight (MW[g mol^−1^]), the number of hydroxyl groups (OH), carbon atom count (C), ring count (RC), molecular volume (A3 [Å^3^]), hydrogen bond donor count (HBD), and hydrogen bond acceptor count (HBA). The specific numerical values assigned to each additive are summarized in Table 1. The inputs for the RDKit calculations were standardized using the canonical 2D topological structures, employing the cyclic pyranose/furanose forms for the monosaccharides and disaccharides, and the open-chain form for the sugar alcohol. The control (pg) was parameterized with zero values across all these molecular features.

**Table 1:** Molecular descriptors implemented for the machine learning characterization of the added saccharide derivatives

Additive type	MW (g mol^−1^)	OH	C	RC	A3 (Å^3^)	HBD	HBA
Sugar-free (pg)	0.00	0	0	0	0	0.0	0.0
Sucrose (suc)	342.30	8	12	2	280.82	8	11
Glucose (glu)	180.16	5	6	1	151.29	5	6
Fructose (fru)	180.16	5	6	1	152.29	5	6
Sorbitol (sor)	182.17	6	6	0	163.23	6	6

The final dataset was constructed by incorporating two macroscopic processing features—concentration and water content—alongside the seven molecular descriptors, yielding a total of nine input variables. The target variable, Young's modulus (*E*), spans a wide dynamic range across several orders of magnitude depending on the sugar type and drying duration. To accommodate this high numerical variance and to suppress exponential scaling biases during the regression process, the target variable was transformed into a logarithmic scale using the natural logarithm function, *y* = ln(*E* + 1), implemented *via* the np.log1p function in NumPy. This specific transformation ensures that the dynamic range is effectively compressed into a well-behaved continuous distribution, while simultaneously avoiding mathematical singularity errors (*i.e.*, divergence to negative infinity) in instances where the modulus approaches zero or exhibits near-zero values in highly compliant states.

To eliminate any data-splitting artifacts, sampling biases, and stochastic dependencies associated with specific random seeds—which are often critical in small-scale experimental datasets—a highly rigorous validation loop was implemented. Model validation was comprehensively conducted using ten distinct random seeds (seeds 0–9), with each seed running an independent 5-fold cross-validation (KFold) scheme with prior random shuffling. This exhaustive configuration yielded a total of 50 independent training and evaluation cycles. Within each fold, out-of-sample predictions were systematically evaluated on the unseen test datasets (*i.e.*, data completely excluded from the model training phase). These predictions were then compiled to construct the observed-versus-predicted plot and to calculate the overall robust mean coefficient of determination (mean *R*^2^ ± SD). To gain mechanistic insights into the feature dependencies, relative feature importances were extracted across the entire 50-cycle validation framework. Both Mean Decrease in Impurity (MDI) and Permutation Importance (PI) profiles were computed for each validation fold. The final relative rankings and historical contributions of the nine features were evaluated as the global mean scores and strict standard deviations (±SD) across all 50 simulated iterations. This multi-seed ensemble approach rigorously guarantees that the captured physicochemical feature hierarchy reflects generalized materials correlations rather than randomized local optimizations.

### 2.8. Determination of mechanical transition points *via* statistical segmented regression

To quantitatively and objectively identify the critical moisture thresholds where the plasticization mechanism shifts, transition-point analysis was implemented on the experimental raw data of Young's modulus *versus* water content for both sucrose- and sorbitol-doped gelatin systems. The exact transition points (break points) were determined automatically from the empirical data points *via* a continuous piecewise linear regression approach using the pwlf library in Python.^26^ The algorithm dynamically optimizes the locations of the line segments by minimizing the sum of squared residuals, thereby capturing the distinct change in the slope where the moisture-induced mechanical decay terminates without introducing subjective human bias. For visual clarity and to present the continuous macroscopic trend of this mechanical transition, a four-parameter logistic regression model was additionally fitted to the dataset using the curve_fit function from the scipy.optimize library and superimposed on the plots as a guide-to-the-eye. These mathematically optimized break points were utilized to rigorously define the boundary where the micro-molecular features of the sugars transition from active water-mediated matrix-disruption to macro-structural saturation.

## 3. Results and discussion

### 3.1. Effect of adding saccharide derivatives on gelatin hydrogels

Firstly, we investigated the gelation temperatures of gelatin solutions containing the various concentration of saccharides, which serve as indicators of the interaction among gelatin networks. Rheological measurements of the gelatin solutions during cooling process can clearly define gelation temperatures as the crossover point where the storage modulus (*G*′) surpasses the loss modulus (*G*″). The measurements revealed that the addition of any saccharide derivatives increased the gelation temperatures compared to the original gelatin solution (Fig. 2). This result indicates that gelatin network formation proceeded at higher temperatures in the presence of saccharides, which is consistent with previous studies.^18–20^ This promoted gelation can be attributed to preferential hydration by the saccharides derivatives and the restriction of free water.^20^ In general, the random-coil structure of gelatin is stabilized through interactions with surrounding water molecules. When saccharides co-existing with gelatin, water molecules preferentially hydrate the hydroxyl groups of the saccharides, resulting in reduced free water surrounding the gelatin chains. Consequently, the stability of the random coil structure is relatively decreased, thereby promoting intermolecular association and helix formation among the gelatin chains even at higher temperatures, leading to an elevated gelation temperature.

**Fig. 2:**
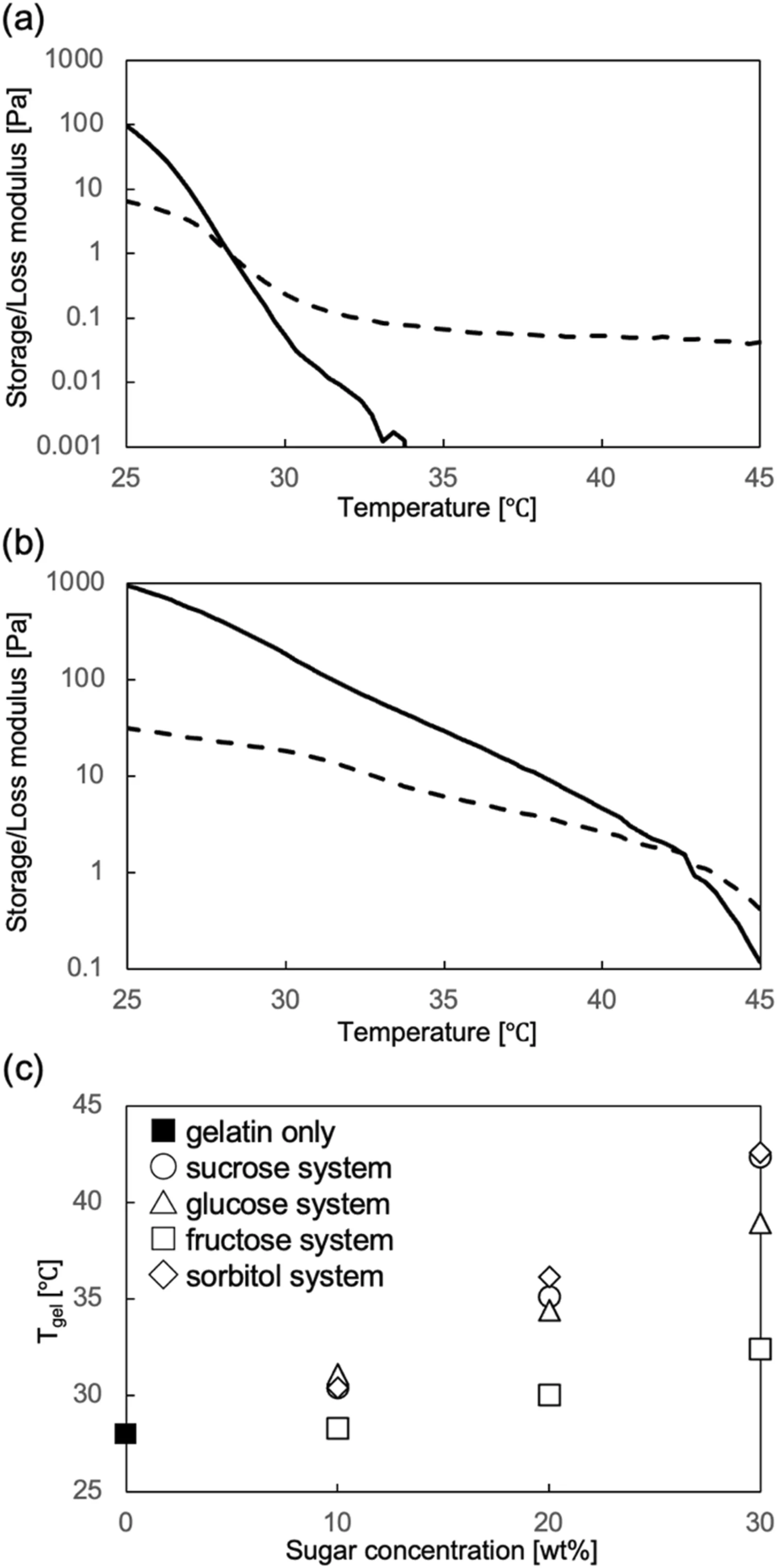
Rheological properties of gelatin solutions during cooling from 45 to 25 °C at a rate of 0.5 °C min^−1^. The storage modulus (solid line) and the loss modulus (dashed line) change in solutions of (a) 10 wt% gelatin (porcine skin) and (b) 10 wt% gelatin with 30 wt%. (c) Gelation temperatures (*T*_gel_) of a series of gelatin solutions containing varied concentration of the saccharides. *T*_gel_ were defined by the crossover point of *G*′ and *G*″. Symbols: ■ gelatin only, ○ sucrose system, △ glucose system, □ fructose system, and ◇ sorbitol system.

The hydration ability of saccharides has been reported to follow the order of sucrose > glucose > fructose,^19^ which is consistent with the order of gelation temperatures described above. The water-binding ability of saccharides, – namely, their ability to exclude free water surrounding gelatin chains – strongly correlated with the gelation behavior of the gelatin hydrogels. Notably, the addition of sorbitol exhibited a pronounced increase in the gelation temperature, comparable to that of the sucrose system. Al though systematic studies remain limited regarding a direct comparisons of the hydration properties of sorbitol with those of other saccharides, sorbitol is known to possess high hydrophilicity derived from its polyol structure.^28,29^ Furthermore, another effect relating to differences in molecular structure can be assumed because sorbitol has a flexible linear structure unlike the other cyclic saccharides. Consequently, not only changes in the hydration state but also differences in molecular arrangement and intermolecular interaction modes near the gelatin chains may have contributed to these gelation behaviors.

### 3.2. Mechanical property of gelatin sheets prepared *via* drying the gelatin hydrogels

When water is abundant, the water-binding property of the added saccharides and sorbitol play a dominant role in determining physical property of the hydrogels. On the other hand, what becomes the dominant factor once the water content is significantly reduced? Here, dried sheets, prepared by extensively removing moisture from the gelatin hydrogels, were utilized to evaluate the relationship between physical properties and the mixed saccharide derivatives under reduced water content. Such dried states are commonly observed in highly concentrated confectionery products such as gummy candies and marshmallows,^18,30–32^ where specific intermolecular interactions driven by the low water content play an important role in determining the bulk physical properties. After one day of water evaporation, the water contents reached 0.056, 0.840, 1.042, 0.904, and 0.833 g per gram of gelatin for pristine gelatin (PG) and the gelatin containing 30 wt% sucrose, glucose, fructose and sorbitol, respectively. Owing to water-binding ability of the saccharide derivatives, the sheet containing them remained slightly higher water contents than the PG sheet. This residual water could impart flexibility to the dried gelatin sheets. Although the PG sheet exhibited brittle fracture upon folding deformation, the sheets containing the saccharides underwent large deformations, showing enhanced flexibility (Fig. 3a). Tensile stress–strain curves further confirmed that the addition of saccharide derivatives significantly improved the strain at break compared to that of the PG sheet (Fig. 3b–f). Notably, this improvement in flexibility cannot be attributed solely to the water contents; the PG sheet with 1.094 g of water per gram of gelatin exhibited only 89% strain, which is much lower than that of the gelatin sheets with saccharide derivatives (Fig. S1), This indicates that saccharide derivatives play a role beyond simply retaining moisture. Furthermore, compared to sucrose, a pronounced increase in elongation was observed in the glucose-, fructose-, and sorbitol-added systems, demonstrating that the mechanical properties of the dried sheets vary substantially depending on the specific type of saccharide incorporated.

**Fig. 3:**
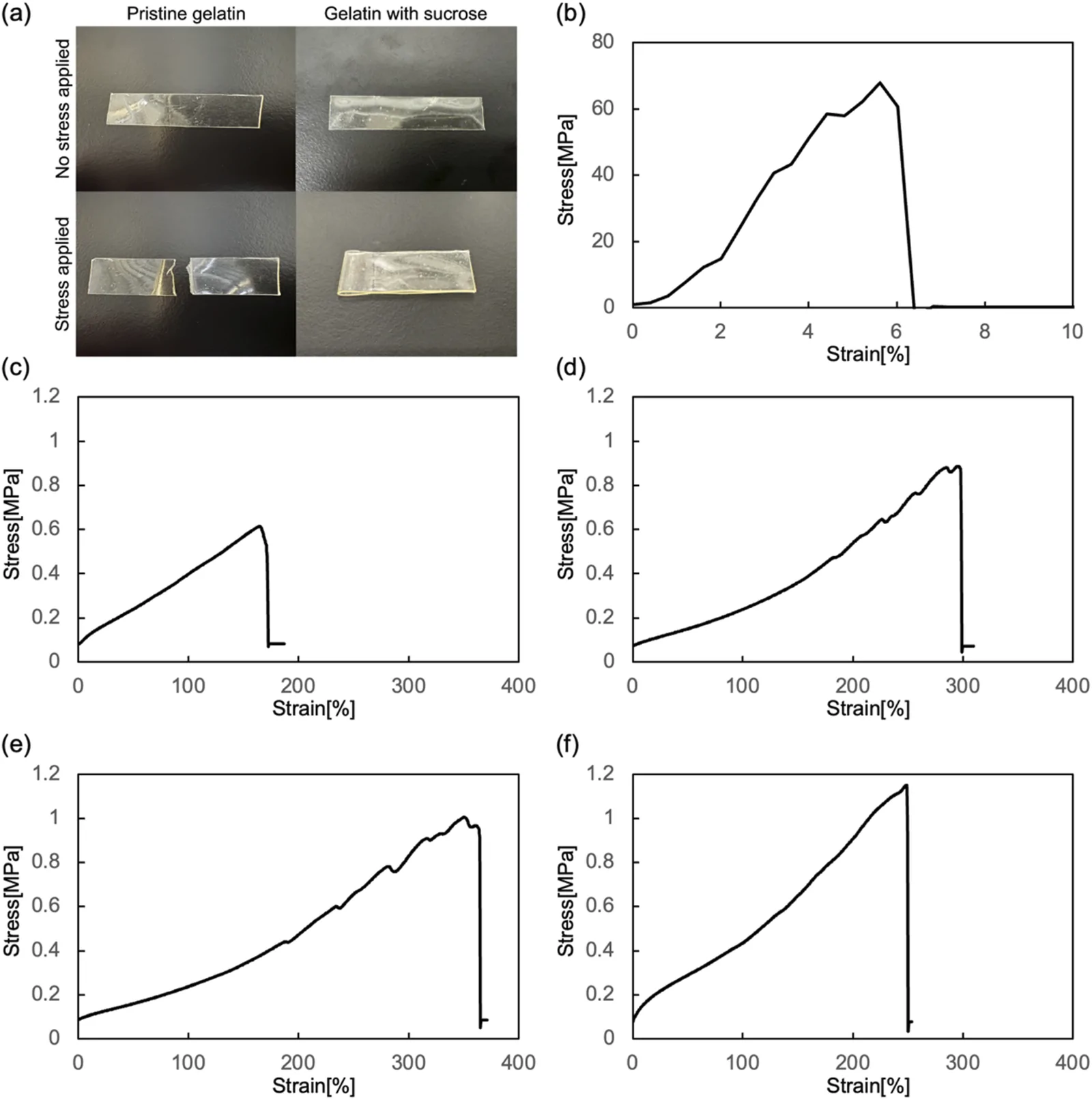
Mechanical property of dried gelatin sheets. (a) Visual observations of sheets before folding (no stress applied) and during folding deformation (stress applied). Tensile stress–strain curves of the (b) PG and gelatin sheets containing 30 wt% (c) sucrose, (d) glucose, (e) fructose, and (f) sorbitol, respectively. All samples were dried at room temperature for 24 h prior to testing.

The physical architecture within the network would be different between the PG and the other gelatin sheets. To elucidate the underlying mechanism of the enhanced flexibility, ATR-FTIR measurements of the sheets were performed (Fig. 4). In all samples, specific absorption bands corresponding to the Amide I band (approximately 1630–1650 cm^−1^) and the Amide II band (approximately 1540–1560 cm^−1^) were clearly observed, which are typical spectra derived from the secondary structure of gelatin. In addition, the saccharide-containing systems exhibited characteristic peaks around 900–1150 cm^−1^ derived from C–O–C and C–OH stretching vibrations, confirming that the saccharides remained within the gelatin matrix even after drying.

**Fig. 4:**
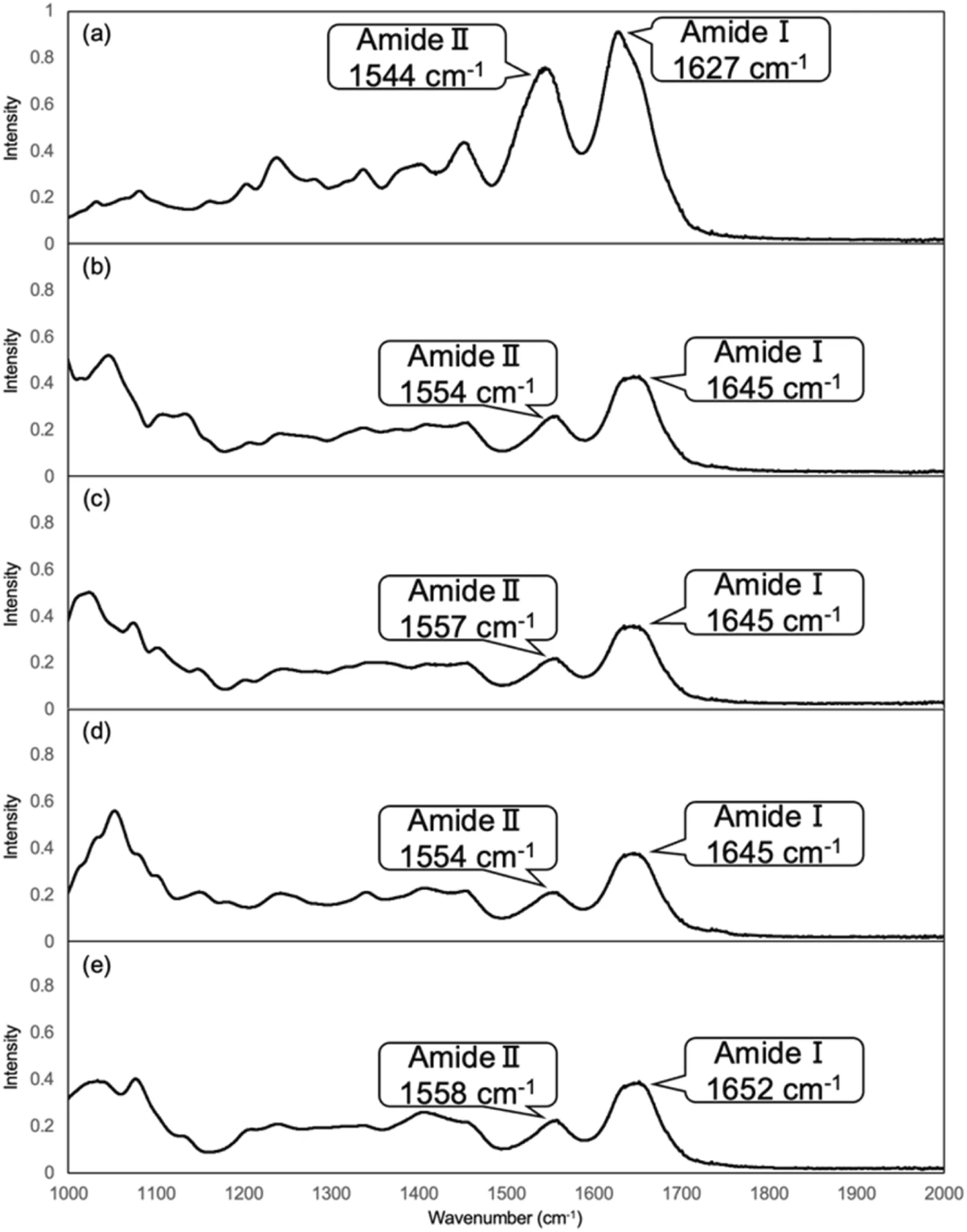
ATR-FTIR spectra of dried gelatin sheets. (a) PG and gelatin sheets containing 30 wt% (b) sucrose, (c) glucose, (d) fructose, and (e) sorbitol after drying at room temperature for 24 h. Characteristic Amide I and Amide II bands associated with the molecular structure and hydrogen-bonding environment of gelatin were observed in all spectra.

The Amide I band mainly corresponds to C=O stretching vibrations and reflects the helical and random coil structures of gelatin.^33^ In the PG sheet, the Amide I band was observed at approximately 1634 cm^−1^ (Fig. 4a), whereas the saccharide-containing sheets exhibited a shift toward higher wavenumber, around 1645 cm^−1^ (Fig. 4b–e). In general, lower-wavenumber absorption of the Amide I band is associated with stronger hydrogen bonding and more ordered triple-helix structures. Therefore, this shift to higher wavenumber suggests that the addition of saccharides altered the hydrogen-bonding environment surrounding the gelatin chains. Similar peak shifts were also observed in the Amide II band (Fig. 4b–e), indicating possible alterations in intermolecular interactions and hydration states under the dried conditions.^33^ These shifts in the Amide II band further support that saccharide addition influenced the hydrogen-bonding network around the gelatin chains in the dried state. Considering the enhanced flexibility and elongation property of the gelatin sheets (Fig. 3), the saccharide derivatives under reduced water contents conditions likely promote the molecular chain mobility within the gelatin network, acting similarly to plasticizers. This behavior is completely opposite to the role that saccharide derivatives play in the gelatin hydrogel state, where they promote hydrogen bonding among gelatin chains and elevate gelation temperature (Fig. 2).^20^

### 3.3. Machine-learning-based identification of regulatory factors for sheet mechanical properties

Under the reduced water contents, the influence on the mechanical properties of the sheets differed among the added saccharide derivatives. In the case of PG sheets, decreased water contents generally increased sheet stiffness, thereby restricting elongation (Fig. 3b and S1). The differences in mechanical behavior among the saccharide derivatives-containing sheets cannot be explained by the water content alone. For instance, the addition of sorbitol (0.833 g g^−1^-gelatin) resulted in a significantly larger strain at break (elongation) compared to the addition of sucrose (0.840 g g^−1^-gelatin) (Fig. 3c and f). Furthermore, although the glucose-added sheet possessed the highest water content (1.042 g g^−1^-gelatin), it did not exhibit a proportional increase in elongation when compared to the fructose-added sheet (Fig. 3d and e). These discrepancies clearly demonstrate that variations in mechanical properties do not depend solely on the amount of residual water; there are other factors to determine the mechanical properties.

To provide deeper insight into the regulatory factors governing the mechanical properties of the sheets, we prepared sheets with varied concentrations of the saccharide derivatives, resulting in different water contents. Here, the Young's modulus of the sheets was used to evaluate the mechanical properties because the aforementioned changes in parameters influence sheet thickness. Since it is highly difficult to completely adjust the water contents in sample, preparing identical replicate samples for traditional statistical analysis is impractical. Therefore, a machine-learning-based multivariate analysis was performed.

Sheet samples with systematically varied additive concentrations and residual water contents were prepared to construct the dataset. In addition, to quantitatively represent the differences among additive species, molecular structural descriptors including the number of hydrogen-bond donors (HBD), hydrogen-bond acceptors (HBA), molecular size (A3), molecular weight (MW), the number of hydroxyl groups (OH), and ring structure count (RC) were introduced as explanatory variables (Table 1). Then, we utilized a machine-learning regressor based on the Random Forest Regressor (RFR) algorithm. To guarantee statistical reproducibility and eliminate data-splitting artifacts often observed in experimental datasets, the model was evaluated *via* a robust multi-seed framework comprising ten distinct random seeds with 5-fold cross-validation, totaling 50 independent simulation cycles.

By training the RFR model on this compiled dataset of 122 experimental matrix points, the model successfully predicted the Young's modulus with a high coefficient of determination (mean *R*^2^ = 0.9739 ± 0.0089), demonstrating exceptional predictive performance and generalization capabilities (Fig. 5). The relative feature importances extracted cross the 50 validation iterations afford crucial mechanistic insights into the mechanical modulation of the gelatin matrices (Fig. 6). In the MDI profile (Fig. 6a), macroscopic processing variables—namely, sugar concentration (63.20% ± 7.36%) and water content (25.79% ± 7.42%)—exerted an overwhelming dominance, collectively accounting for the vast majority of the total predictive power (totaling 89.03%). This primary dependence underscores that the macro-structural network density and the degree of plasticization induced by residual moisture are the paramount determinants of the sheet's Young's modulus. On the other hand, parameters associated with the structural characteristics of the additives also exhibited non-negligible importance scores. Among the microscopic molecular descriptors in the MDI profile, the molecular volume (A3: 2.14% ± 0.19%) emerged as the most influential feature, slightly surpassing specific hydrogen-bonding metrics such as HBD (1.90% ± 0.22%) and OH (1.90% ± 0.20%), while the ring count (RC) resided at the absolute bottom (0.30% ± 0.08%).

**Fig. 5:**
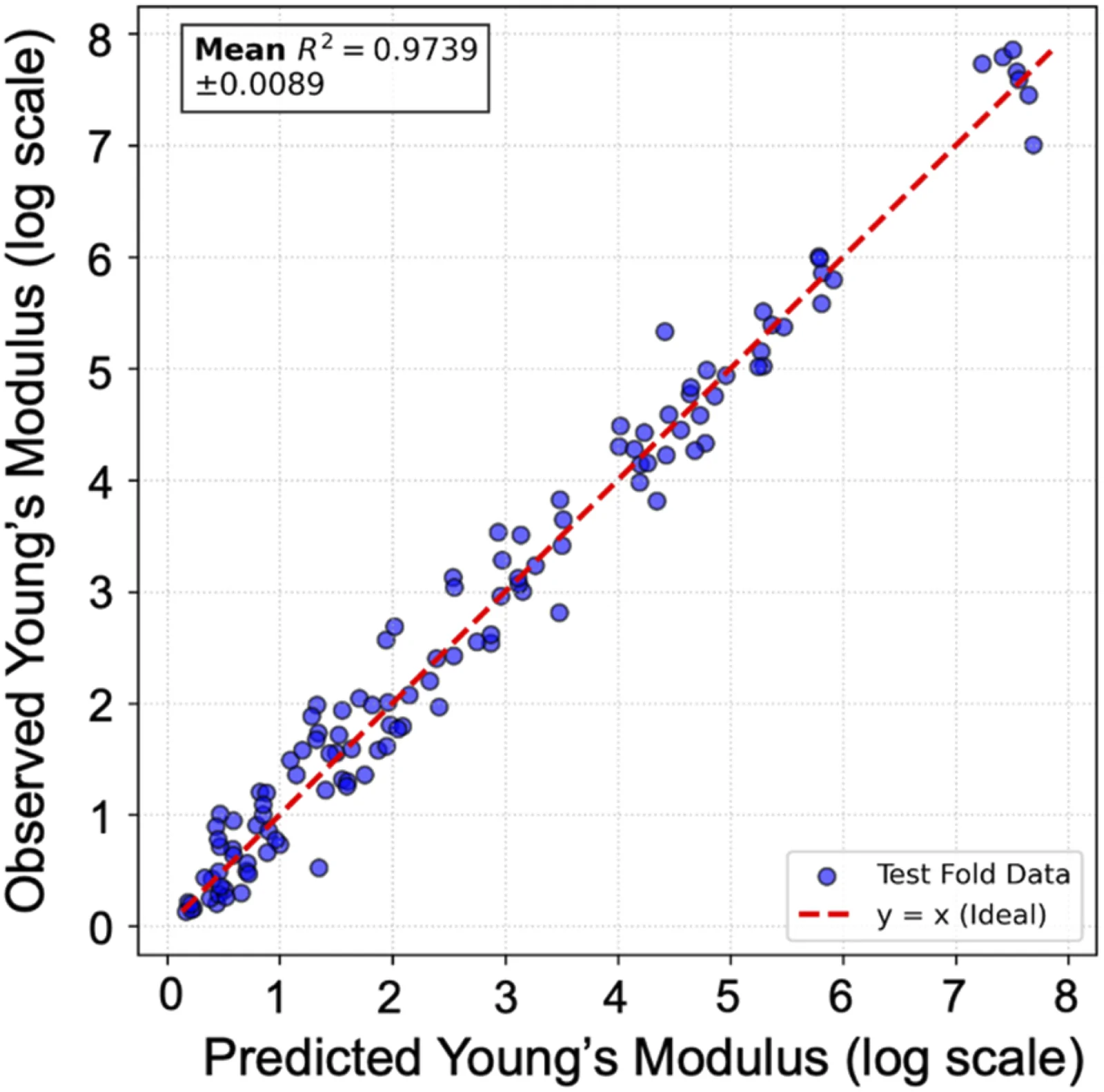
Observed *vs.* predicted Young's modulus of sugar-doped gelatin matrices. The scatter plot demonstrates the prediction performance evaluated 10-seed 5-fold cross-validation framework, totaling 50 independent simulation cycles. The target variable is transformed into a logarithmic scale as ln(*E* + 1) to compress the wide dynamic range. The robust mean coefficient of determination (mean *R*^2^ = 0.9739 ± 0.0089) derived across all 50 validation folds is superimposed on the plot. The dashed red line represents the ideal prediction (*y* = *x*).

**Fig. 6:**
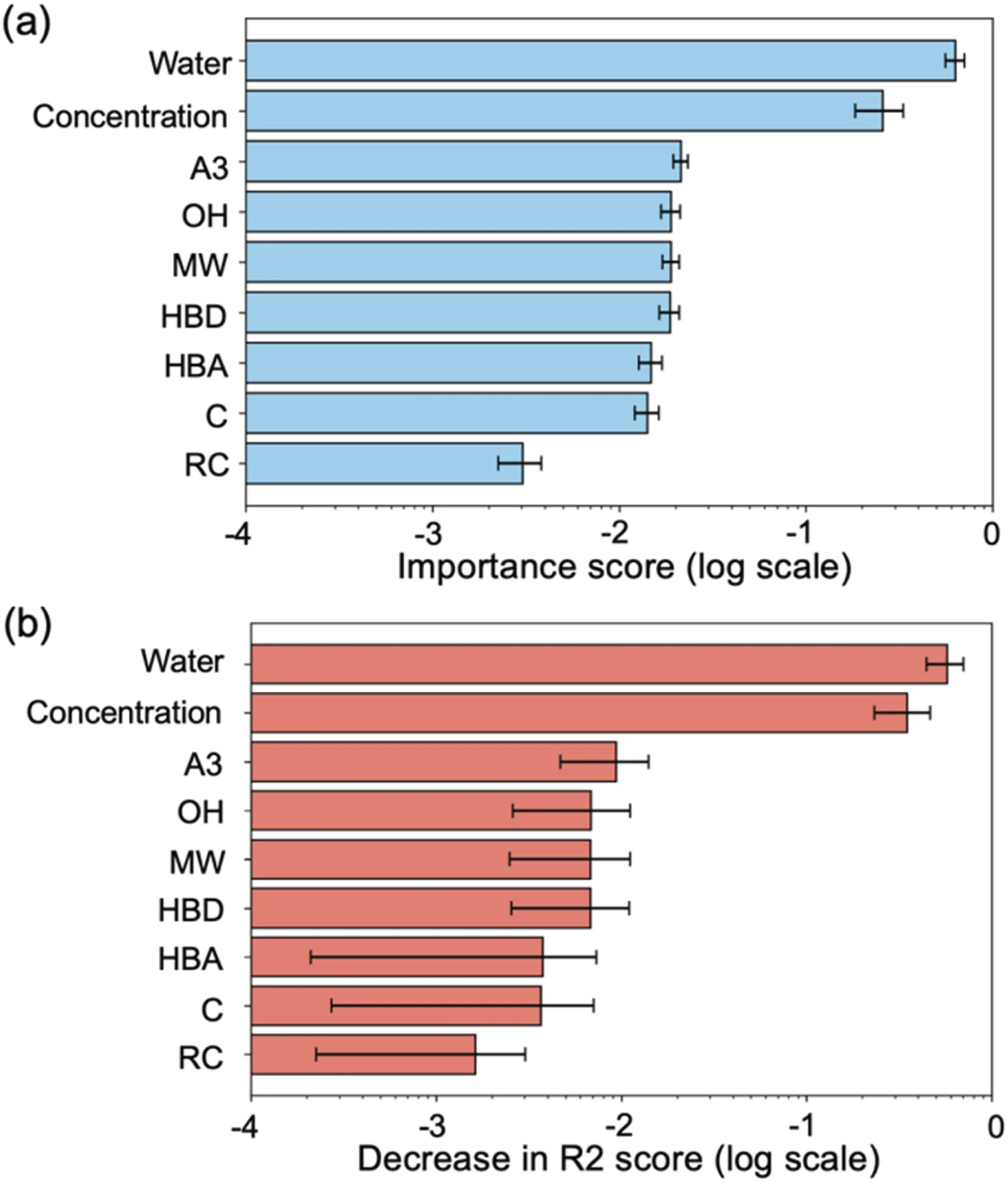
Robust feature importance profiles for predicting the Young's modulus of gelatin sheets. Profiles are evaluated *via* (a) Mean Decrease in Impurity (MDI) and (b) Permutation Importance (PI) based on a 10-seed 5-fold cross-validation framework (50 independent cycles total). The horizontal bars and error bars denote the global mean scores and standard deviations (±SD) calculated across all 50 validation iterations. The importance scores are displayed on a logarithmic scale to clearly resolve the micro-structural descriptors against the overwhelmingly dominant macro-environmental factors. The evaluated descriptors include macroscopic processing features (concentration and water content) along with molecular features (MW, OH, C, RC, A3, HBD, and HBA).

Crucially, the robust PI profile (Fig. 6b), which evaluates the model's generalization sensitivity on unseen test data, confirmed a highly converged and consistent feature dependency. In the PI profile, sugar concentration (57.50% ± 12.97%) and water content (34.92% ± 11.65%) shared the primary contribution, while molecular volume (A3: 0.92% ± 0.47%) and hydrogen-bonding metrics (HBD: 0.68% ± 0.43%; OH: 0.67% ± 0.43%) strictly maintained their co-dominant positions among the chemical descriptors. Although the standard deviation of the microscopic descriptors such as A3 appears relatively wide compared to their mean scores, this numerical variance accurately reflects the localized sample heterogeneity inherent to experimental datasets across different validation folds. Most notably, through the exhaustive 50-cycle validation framework, the previously observed high numerical variance in the A3 importance score was completely stabilized, and the entire feature hierarchy converged tightly with no relative order inversions. This remarkable statistical stability firmly demonstrates that the captured structure–property relationship is highly reproducible and represents a generalized physicochemical correlation rather than a randomized local optimization.

From a materials science perspective, this striking convergence underscores that the microscopic modulation of the matrix modulus is governed by a highly reproducible, dual-action physicochemical mechanism: the steric hindrance effect driven by spatial bulkiness (represented by A3) and the localized reinforcement of the intermolecular network *via* dense hydrogen-bonding functionalities (represented by OH). Although several molecular descriptors (*e.g.*, MW, A3, OH, and HBD) exhibit mutual correlations due to the inherent chemical structure of saccharides, the rigorous 50-cycle cross-validation framework demonstrated high stability in feature rankings with tight standard deviations. This statistical convergence indicates that the model does not merely switch arbitrarily between redundant descriptors across different folds. Instead, it identifies spatial volume hindrance (represented by A3) and interactive hydrogen-bonding density (represented by OH/HBD) as cooperative triggers that jointly dictate the local chain mobility within the matrix. Consequently, this micro-governing mechanism would provide a robust thermodynamic explanation for the distinct gap between the plasticization transition points of sorbitol and sucrose observed in Fig. 7, validating that bulk molecular sizes and interactive site densities are the core parameters driving the macroscopic property transitions.

**Fig. 7:**
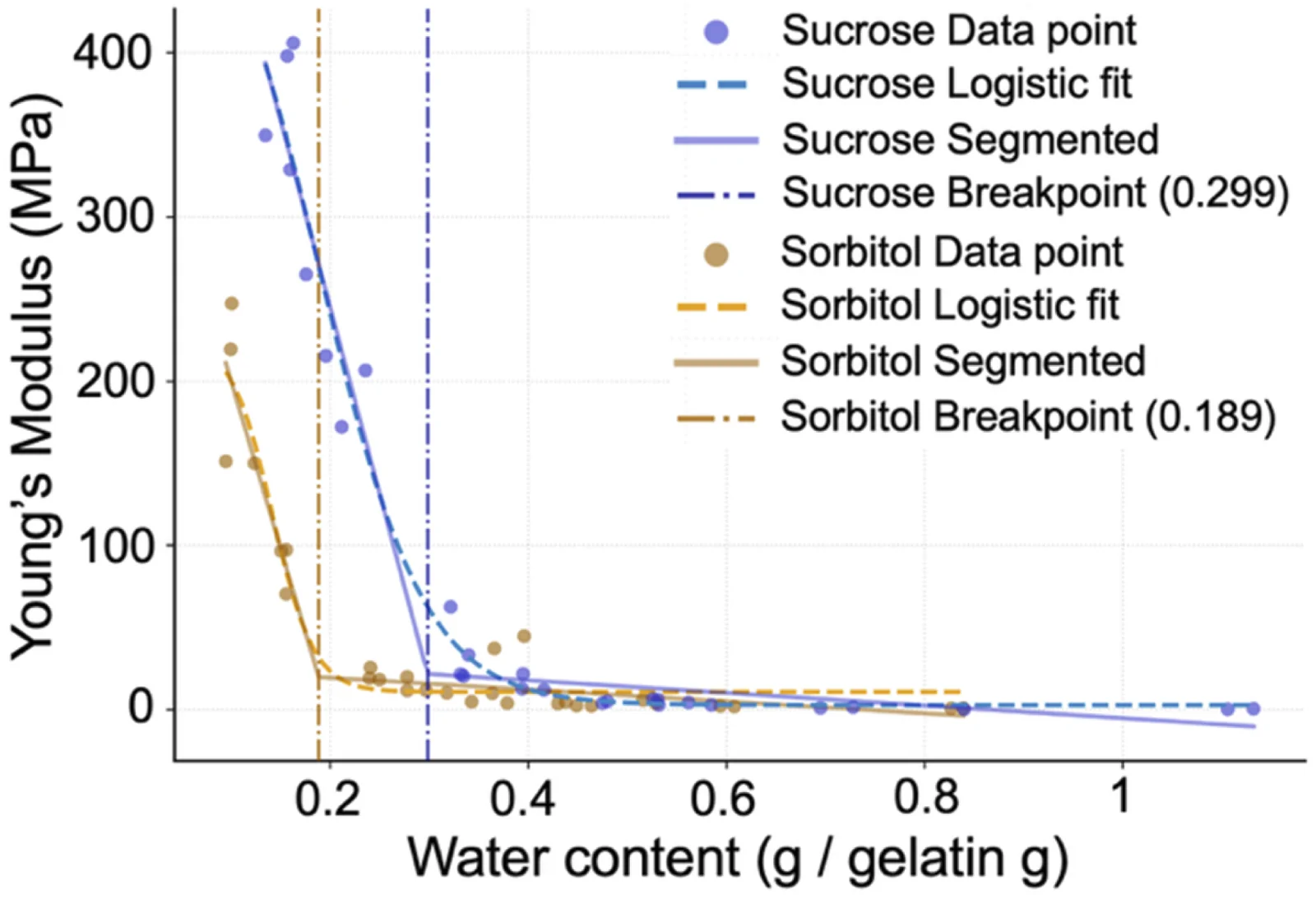
Experimentally observed and mathematically modeled Young's modulus as a function of water content for sucrose- and sorbitol-doped gelatin systems. The scatter plots represent the empirical data points for sucrose (purple circles) and sorbitol (brown circles). The solid lines depict the piecewise linear regression models optimized *via* the pwlf library, automatically identifying the critical mechanical transition points (break points). The mathematically determined break points are marked by the vertical dash-dotted lines for sorbitol (brown, left) and sucrose (light blue, right). To provide a continuous macroscopic trend and serve as a guide-to-the-eye, four-parameter logistic regression curves (dashed lines; sucrose logistic and sorbitol logistic) are superimposed on the respective datasets.

## 4. Conclusion

We investigated the effect of saccharide derivative addition on gelatin-based structures, spanning from hydrogels to dry sheets. Among the various saccharide derivatives tested, the addition of sucrose and sorbitol exhibited a significant increase in the gelation temperature of gelatin solutions, this was attributed to favorable water-binding properties of them, which enhance inter- and intramolecular interactions among gelatin chains, consistent with previous studies. Conversely, in the sheet state under significantly reduced water contents from hydrogel, the addition of saccharide derivatives increased the extensibility of the sheets. ATR-FTIR spectra suggest the addition of saccharide derivatives disrupted these inter- and intramolecular interactions among the gelatin chains to increase their motility, thereby acting like plasticizers. Random Forest Regression (RFR) analysis revealed that not only the water content and the concentrations but also the chemical nature of the saccharide derivatives—including molecular volume (A3) and hydrogen-bond functionalities (HBD and OH)—exhibited varying degrees of feature importance in determining the mechanical properties of the sheets. Notably, gelatin sheets with sorbitol required a significantly smaller water content to achieve comparable properties than those with sucrose, a distinction that was not observed in the hydrogel state. These insights open new avenues for the rational design of green and sustainable gelatin-based biomaterials with precisely tuned mechanical profiles.

## Author contributions

G. Y.: investigation, writing – original draft, funding acquisition, M. M.: data curation, methodology, funding acquisition, writing – review & editing, S. O.; conceptualization, funding acquisition, supervision, writing – review & editing.

## Conflicts of interest

No conflicts of interest.

## Supplementary Material

RA-016-D6RA06070F-s001

RA-016-D6RA06070F-s002

## Data Availability

The data that support the findings of this study are available from the corresponding authors [M. M., and S. O.], upon reasonable request. Supplementary information (SI): Fig. S1; tensile strength test of PG film containing 1.094 g water per gram of gelatin, Table S1: various gelatin films using as leraning data for random forest regression, Table S2: mean decrease impurity (MDI) for each of the 50 runs (10 random seeds × 5-fold CV) in Section 2.7 process, Table S3: permuation importance (PI) for each of the 50 runs (10 random seeds × 5-fold CV) in Section 2.7 process, and Python code using Sections 2.7 and 2.8. See DOI: https://doi.org/10.1039/d6ra06070f.
