# Supplementary material for: Machine-learning-driven rational design of gelatin architectures: saccharide derivatives as structural plasticizers under low water content
Source: RSC Adv. 2026 Sep 15;16(46):59311–9. doi: 10.1039/d6ra06070f (PMC13575754; doi:10.1039/d6ra06070f)
Supplement: Supplementary file 1 [file RA-016-D6RA06070F-s002.pdf]

Supplementary Information (SI) for RSC Advances.
This journal is © The Royal Society of Chemistry 2026

Machine-Learning-Driven Rational Design of Gelatin Architectures: Saccharides Derivatives as Structural Plasticizers under Low Water Content

Gaku Yazawa[1], Masato Masuda[2], and Shigehito Osawa[1,3]

[1]Department of Biomedical Engineering, Graduate School of Life Science, Toyo University.
48-1 Oka, Asaka, Saitama, 351-8510, Japan, osawa@toyo.jp
[2]Department of Information Science and Art, Faculty of Information Science and Art, Toyo University.
2100 Kujirai, Kawagoe, Saitama, 350-8585, Japan, masuda061@toyo.jp
[3]Department of Biomedical Engineering, Faculty of Life Science, Toyo University.
48-1 Oka, Asaka, Saitama, 351-8510, Japan

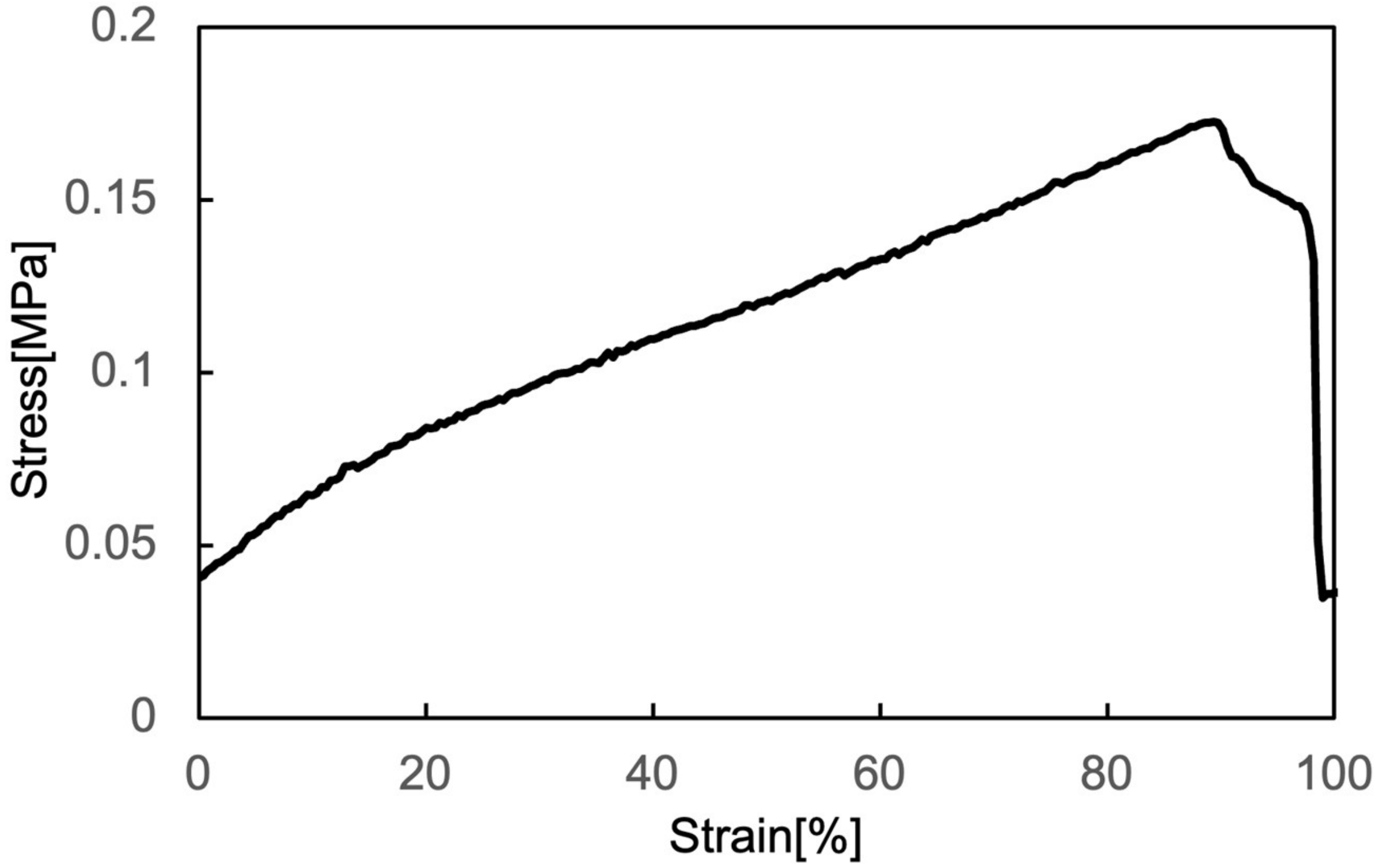

Figure S1 Tensile strength test of PG film containing 1.094 g water per gram of gelatin

Table S1 Various gelatin films using as learning data for random forest regression

Sample entry	Saccharide amount (g)	Water amount /gram of gelatin (g)	Young Modulus (MPa)
Suc10-1<br>Suc10-2	0.50<br>0.50	0.322<br>0.212	62.57<br>172.04
Suc10-3	0.50	0.236	206.49
Suc10-4	0.50	0.176	264.89
Suc10-5	0.50	0.196	215.36
Suc10-6	0.50	0.157	397.74
Suc10-7	0.50	0.160	328.58
Suc10-8	0.50	0.135	349.47
Suc10-9	0.50	0.163	405.61
Suc20-1	1.00	0.562	4.35
Suc20-2	1.00	0.480	5.10
Suc20-3	1.00	0.526	6.74
Suc20-4	1.00	0.394	12.70
Suc20-5	1.00	0.416	12.07
Suc20-6	1.00	0.332	21.68
Suc20-7	1.00	0.335	20.50
Suc20-8	1.00	0.340	33.34
Suc20-9	1.00	0.395	21.87
Suc30-1	1.50	0.840	0.35
Suc30-2	1.50	1.106	0.33
Suc30-3	1.50	1.132	0.63
Suc30-4	1.50	0.695	1.17
Suc30-5	1.50	0.728	1.58
Suc30-6	1.50	0.475	3.91
Suc30-7	1.50	0.531	6.27
Suc30-8	1.50	0.532	2.90
Suc30-9	1.50	0.585	2.90
Glu10-1	0.50	0.359	24.42
Glu 10-2	0.50	0.335	32.39
Glu 10-3	0.50	0.228	52.69
Glu 10-4	0.50	0.208	61.75
Glu 10-5	0.50	0.194	87.67
Glu 10-6	0.50	0.179	82.96
Glu 10-7	0.50	0.143	117.39
Glu 10-8	0.50	0.146	124.38
Glu 10-9	0.50	0.130	146.01
Glu 20-1	1.00	0.661	0.94
Glu 20-2	1.00	0.663	1.47
Glu 20-3	1.00	0.472	3.43
Glu 20-4	1.00	0.570	2.33
Glu 20-5	1.00	0.398	5.59
Glu 20-6	1.00	0.347	4.58
Glu 20-7	1.00	0.305	13.68
Glu 20-8	1.00	0.311	6.14
Glu 20-9	1.00	0.335	6.26
Glu 20-10	1.00	0.320	6.44
Glu 30-1	1.50	1.051	0.16

Glu 30-2	1.50	1.033	0.22
Glu 30-3	1.50	0.756	0.29
Glu 30-4	1.50	0.720	0.55
Glu 30-5	1.50	0.563	0.64
Glu 30-6	1.50	0.623	0.76
Glu 30-7	1.50	0.504	1.74
Glu 30-8	1.50	0.521	1.45
Glu 30-9	1.50	0.558	1.05
Glu 30-10	1.50	0.543	1.18
Fru10-1	0.50	0.302	29.45
Fru 10-2	0.50	0.336	15.70
Fru 10-3	0.50	0.196	44.30
Fru 10-4	0.50	0.206	71.15
Fru 10-5	0.50	0.169	67.44
Fru 10-6	0.50	0.199	72.70
Fru 10-7	0.50	0.118	139.15
Fru 10-8	0.50	0.128	115.25
Fru 10-9	0.50	0.141	75.04
Fru 10-10	0.50	0.157	84.72
Fru 20-1	1.00	0.582	1.08
Fru 20-2	1.00	0.680	1.73
Fru 20-3	1.00	0.420	3.85
Fru 20-4	1.00	0.680	1.36
Fru 20-5	1.00	0.343	2.67
Fru 20-6	1.00	0.354	2.73
Fru 20-7	1.00	0.227	8.01
Fru 20-8	1.00	0.296	6.97
Fru 20-9	1.00	0.283	5.01
Fru 20-10	1.00	0.326	3.87
Fru 30-1	1.50	0.843	0.18
Fru 30-2	1.50	0.965	0.24
Fru 30-3	1.50	0.722	0.23
Fru 30-4	1.50	1.088	0.14
Fru 30-5	1.50	0.528	0.30
Fru 30-6	1.50	0.562	0.43
Fru 30-7	1.50	0.399	0.69
Fru 30-8	1.50	0.489	0.60
Fru 30-9	1.50	0.556	0.53
Fru 30-10	1.50	0.510	0.39
Sor10-1	0.50	0.366	37.29
Sor 10-2	0.50	0.396	44.80
Sor 10-3	0.50	0.156	70.43
Sor 10-4	0.50	0.151	96.64
Sor 10-5	0.50	0.156	97.29
Sor 10-6	0.50	0.101	247.16
Sor 10-7	0.50	0.100	219.31
Sor 10-8	0.50	0.124	150.04
Sor 10-9	0.50	0.095	151.14
Sor 20-1	1.00	0.516	5.93
Sor 20-2	1.00	0.529	3.75
Sor 20-3	1.00	0.318	10.30
Sor 20-4	1.00	0.364	10.08

Sor 20-5	1.00	0.278	11.67
Sor 20-6	1.00	0.297	11.81
Sor 20-7	1.00	0.240	19.21
Sor 20-8	1.00	0.241	25.64
Sor 20-9	1.00	0.250	18.32
Sor 20-10	1.00	0.278	19.95
Sor 30-1	1.50	0.839	1.00
Sor 30-2	1.50	0.827	0.89
Sor 30-3	1.50	0.594	2.31
Sor 30-4	1.50	0.608	1.98
Sor 30-5	1.50	0.464	2.40
Sor 30-6	1.50	0.449	2.53
Sor 30-7	1.50	0.343	4.88
Sor 30-8	1.50	0.379	4.02
Sor 30-9	1.50	0.438	4.68
Sor 30-10	1.50	0.430	3.72
PG-1	0.00	0.056	1105.40
PG-2	0.00	0.014	1728.66
PG-3	0.00	0.068	2285.62
PG-4	0.00	0.014	2119.84
PG-5	0.00	0.023	2589.18
PG-6	0.00	0.118	1980.03
PG-7	0.00	0.113	2420.23

Sample names are defined by amount and species of saccharide derivatives (Suc: Sucrose, Glu: Glucose, Fru: Fructose, Sor: Sorbitol). For example, Suc10 is the name of film prepared from solutions containing gelatin 10 wt% and sucrose 10 wt%. PG is the film prepared without adding saccharide derivatives.

Table S2. Mean Decrease in Impurity (MDI) for each of the 50 Runs (10 Random Seeds × 5-Fold CV) in *Section 2.7* process.

	MW	OH	C	RC	A3	HBD	HBA	Concentration	Water content
0_0	0.01530036	0.01858262	0.01443644	0.00243623	0.02031581	0.01854231	0.0139617	0.61812657	0.27829796
0_1	0.01789499	0.02147743	0.01708907	0.00294467	0.02236759	0.02170601	0.01584692	0.67330047	0.20737284
0_2	0.01952536	0.02403668	0.01832674	0.00340962	0.02277192	0.02237462	0.0173	0.57879932	0.29345575
0_3	0.01554079	0.01855709	0.01327713	0.00348535	0.02059704	0.01875956	0.01302944	0.72372478	0.17302882
0_4	0.01460041	0.01770409	0.01336777	0.00319641	0.01891382	0.01736701	0.01168359	0.63201292	0.271154
1_0	0.01788353	0.01911026	0.01417712	0.00270468	0.02005614	0.01916208	0.01474004	0.62991106	0.2622551
1_1	0.01883606	0.02017275	0.01334471	0.00269532	0.0203809	0.01969431	0.01402274	0.67973913	0.21111407
1_2	0.01768867	0.01823334	0.01479411	0.00609102	0.02040768	0.01868963	0.01532058	0.71507695	0.17369803
1_3	0.01803689	0.01966502	0.01264721	0.00219263	0.02155893	0.0199911	0.01318589	0.60411063	0.28861169
1_4	0.01854107	0.02040179	0.01610493	0.00236705	0.02056001	0.0203192	0.01617997	0.57423169	0.31129431
2_0	0.01714844	0.01758707	0.01188804	0.00315271	0.02066225	0.01799232	0.01350093	0.49779053	0.40027772
2_1	0.0184231	0.01914375	0.01401573	0.00349378	0.02027336	0.01927715	0.01643235	0.60666822	0.28227256
2_2	0.01452708	0.01507637	0.00930349	0.00318929	0.01753929	0.01433888	0.00983722	0.7572625	0.15892587
2_3	0.02092415	0.02223114	0.01460504	0.00231119	0.0238015	0.02251738	0.01802001	0.62421566	0.25137394
2_4	0.01962583	0.02163855	0.01440504	0.00242617	0.02306912	0.02187214	0.01823355	0.52025267	0.35847693
3_0	0.01905281	0.02062708	0.01425668	0.00223743	0.02076693	0.02078516	0.01582055	0.67878476	0.2076686
3_1	0.01905187	0.02043817	0.01612907	0.00391146	0.02042191	0.02060267	0.01826743	0.75066434	0.13051307
3_2	0.01601717	0.01657121	0.01240856	0.00315616	0.01872051	0.01760203	0.01356696	0.6481024	0.253855
3_3	0.01581833	0.01689502	0.01013449	0.00262563	0.01943532	0.01771043	0.0111173	0.58658995	0.31967353

3_4	0.02028211	0.02166613	0.01493368	0.00287888	0.02340072	0.02275264	0.01661274	0.45651645	0.42095666
4_0	0.01551346	0.01546338	0.01136374	0.00318296	0.01875287	0.01642742	0.0134612	0.67300247	0.2328325
4_1	0.01853668	0.01784244	0.01298559	0.00286549	0.02034717	0.01881302	0.01446394	0.60089903	0.29324664
4_2	0.02273194	0.0210547	0.01664859	0.00269169	0.02464469	0.02259655	0.01872735	0.60287327	0.26803122
4_3	0.01958559	0.01898478	0.0130564	0.00256926	0.02202021	0.02021538	0.01514124	0.68250645	0.2059207
4_4	0.0177516	0.01721389	0.01263139	0.00376832	0.01951815	0.0183244	0.01383098	0.61416063	0.28280064
5_0	0.02083439	0.01938333	0.01357795	0.00219785	0.02378578	0.02111417	0.01513735	0.62970635	0.25426284
5_1	0.01902836	0.01885666	0.01350637	0.00337028	0.02236035	0.01972973	0.01592459	0.64088966	0.246334
5_2	0.02084175	0.0195257	0.01478517	0.00491668	0.02460979	0.02083917	0.01691415	0.62871908	0.2488485
5_3	0.01583836	0.01396615	0.00874938	0.00332989	0.01995276	0.01452532	0.01001785	0.57891711	0.33470318
5_4	0.02116839	0.01920224	0.01522781	0.00179623	0.02503869	0.02166824	0.01696172	0.72730172	0.15163495
6_0	0.018251	0.0171212	0.01375951	0.00281392	0.0186127	0.01537326	0.01252572	0.39446469	0.50707799
6_1	0.01942796	0.0195877	0.01504355	0.00276453	0.02048063	0.01725734	0.0134297	0.69805215	0.19395644
6_2	0.0211601	0.01961368	0.01613126	0.00348933	0.01988161	0.01582199	0.0151801	0.65552806	0.23319388
6_3	0.0222309	0.02015952	0.01525801	0.00246996	0.02326191	0.01806153	0.01383071	0.62132249	0.26340497
6_4	0.02209235	0.02049963	0.01653112	0.00440492	0.0230898	0.01807238	0.01505442	0.70451779	0.17573758
7_0	0.02264863	0.01948986	0.01501756	0.00291871	0.0216426	0.01746302	0.01475055	0.67628522	0.20978384
7_1	0.02046156	0.01744532	0.01347226	0.00298982	0.01831483	0.01538591	0.01408823	0.50305468	0.39478739
7_2	0.02320287	0.02000173	0.01488282	0.00232462	0.02160085	0.01751803	0.01492938	0.56671013	0.31882957
7_3	0.02156637	0.01878152	0.0145741	0.00261751	0.02046024	0.01671684	0.01568187	0.66195271	0.22764885
7_4	0.02132662	0.01897017	0.01411701	0.0034476	0.02150458	0.01654141	0.0136088	0.74492565	0.14555817

8_0	0.01406933	0.0152863	0.01076348	0.00298841	0.0184701	0.01550715	0.01150494	0.63863074	0.27277956
8_1	0.01605949	0.01853815	0.01360803	0.0034264	0.02170113	0.01790055	0.01501005	0.69001742	0.20373877
8_2	0.01889063	0.02111713	0.01642246	0.00272615	0.02408743	0.02179308	0.01756563	0.59593837	0.28145913
8_3	0.01804442	0.01957888	0.01546615	0.00287743	0.02220743	0.01906968	0.01710844	0.63470517	0.25094239
8_4	0.01770723	0.01986763	0.01628077	0.00222062	0.022408	0.02040592	0.01808797	0.6574733	0.22554854
9_0	0.01969893	0.02006592	0.01661683	0.00307073	0.0246434	0.02170131	0.01546632	0.54551107	0.33322548
9_1	0.01797868	0.01746676	0.01627752	0.00325472	0.02278868	0.0192878	0.01510315	0.74809354	0.13974915
9_2	0.01943152	0.01971329	0.01747785	0.00158664	0.02400282	0.02120493	0.01653701	0.65425379	0.22579216
9_3	0.01865599	0.01781883	0.0148929	0.00299476	0.02403078	0.02035379	0.01448384	0.63605264	0.25071647
9_4	0.01538404	0.01531912	0.00961033	0.00500024	0.01950097	0.0168724	0.01082987	0.63799463	0.2694884

Table S3. Permutation Importance (PI) for each of the 50 Runs (10 Random Seeds × 5-Fold CV) in *Section 2.7* process.

	MW	OH	C	RC	A3	HBD	HBA	Concentration	Water content
0_0	0.01084329	0.01179312	0.00758868	0.00316197	0.01586118	0.01276094	0.00777429	0.59645117	0.35899416
0_1	0.00397474	0.0047396	0.00119649	0.00070475	0.00581704	0.00509917	0.00118365	0.66229784	0.39029367
0_2	0.00110062	0.00103675	0.00075092	-0.0001365	0.00253086	0.00090201	0.00071725	0.42940356	0.30129441
0_3	0.00287364	0.00343127	0.00105744	0.00087345	0.00643151	0.00377269	0.00076843	0.82027534	0.22784441
0_4	0.00811662	0.01059338	0.0096626	0.00133387	0.01316489	0.01128939	0.00732253	0.57886448	0.31654139
1_0	0.0044486	0.00478971	0.00082525	0.00186937	0.00708706	0.00484625	0.00080279	0.55621345	0.25101245
1_1	0.00451246	0.00580651	0.00519659	-2.06E-05	0.01042731	0.00540221	0.00538553	0.54360155	0.30238273
1_2	0.00550384	0.00648556	0.00464513	-0.0004635	0.00547876	0.00638373	0.0041659	0.82109556	0.1479814
1_3	0.00902226	0.01068528	0.00718381	0.00155583	0.01026963	0.01106835	0.00765582	0.48509772	0.27611667
1_4	0.00926975	0.00891363	0.00287742	0.00315422	0.01068099	0.00916918	0.00285449	0.40298991	0.64813589
2_0	0.00793278	0.0084597	0.00670664	0.00099252	0.0098028	0.00900854	0.00778021	0.42046037	0.43220242
2_1	0.0041495	0.00354825	-0.0012475	0.00286014	0.00883222	0.00424326	-0.0018077	0.620246	0.42138087
2_2	0.0090833	0.00934598	0.00676054	0.00143	0.01145759	0.00894562	0.00676592	0.77413987	0.20893401
2_3	0.00315329	0.00323831	0.0016464	0.00146389	0.00999108	0.0034645	0.00145139	0.47541707	0.45089517
2_4	0.00836863	0.00904078	0.00205743	0.00191069	0.01445027	0.00903113	0.00185268	0.36935117	0.60954942
3_0	0.01078643	0.01082485	0.00610923	0.00349692	0.01338271	0.01027922	0.00647787	0.63961924	0.29647765
3_1	0.00476162	0.00506109	0.0015142	0.00220384	0.00737365	0.00501905	0.00161739	0.63943435	0.2842491
3_2	0.01153274	0.01130741	0.00465117	0.00203949	0.01377963	0.01263163	0.00529633	0.58647004	0.28236893
3_3	0.0048626	0.00511665	0.0046459	0.0004489	0.00508954	0.00593677	0.0055675	0.35037753	0.25688501

3_4	-0.0030533	-0.0032757	-0.0050511	0.00019951	-0.0010479	-0.0029084	-0.0053664	0.27214029	0.56982997
4_0	0.01211067	0.01260673	0.0070992	0.0020725	0.01468836	0.01299029	0.00897613	0.61557428	0.27858545
4_1	-0.0024228	-0.0024211	-0.0038313	0.00106258	-0.0015383	-0.0017671	-0.0041873	0.48792055	0.49014399
4_2	0.00554571	0.0050411	0.00088169	0.00156051	0.00904618	0.00546727	0.00121799	0.4763049	0.40418697
4_3	0.00455691	0.00423669	0.00573456	0.00119623	0.00812583	0.00452542	0.00664361	0.68601551	0.32559624
4_4	0.00873733	0.00845577	0.00661863	0.00036921	0.01075436	0.00885019	0.00688312	0.57170462	0.23472332
5_0	0.00626667	0.00554306	0.00201441	0.00305395	0.01150201	0.00632045	0.00149465	0.58699529	0.41907335
5_1	0.00402454	0.00387774	-0.0018403	0.00103637	0.00539648	0.00337336	-0.0019433	0.58880669	0.26207245
5_2	0.00633921	0.00670152	0.00183491	0.00035756	0.00998591	0.00757326	0.00261126	0.71451997	0.35645131
5_3	0.00842114	0.00675266	0.00512095	0.00188966	0.01011922	0.00704066	0.0061479	0.44905373	0.32830899
5_4	0.0085106	0.00782726	0.00432515	0.00348931	0.01450368	0.00948432	0.00367269	0.68044262	0.38507041
6_0	0.00843497	0.00790991	0.00291543	0.00223468	0.01248456	0.00753543	0.00275996	0.34289791	0.66608803
6_1	0.0043602	0.00468773	0.00169782	0.00167537	0.00543397	0.0039603	0.00158726	0.6145356	0.33839294
6_2	0.01035141	0.00956765	0.00609776	0.00094695	0.00970047	0.00787657	0.00592404	0.59000033	0.34399584
6_3	0.00930052	0.00754778	0.00611663	0.00210428	0.00720545	0.00732197	0.0055189	0.57835869	0.30714804
6_4	0.00685914	0.00647439	0.00569034	0.00247378	0.00755713	0.00531924	0.00502426	0.84859544	0.21851592
7_0	0.00897441	0.00769266	0.00453796	0.0008498	0.00817484	0.00656364	0.00469393	0.60831767	0.2861184
7_1	0.01064996	0.00958473	0.0065244	0.00366597	0.01402779	0.00886125	0.0069051	0.39014516	0.45525045
7_2	0.0040635	0.00368549	-0.0004496	-0.0005279	0.00760518	0.00397295	-0.0001037	0.53201525	0.47268881
7_3	0.01054317	0.00995603	0.00615229	0.00195221	0.01120742	0.00886949	0.00687246	0.6731016	0.28672708
7_4	0.00561952	0.00491081	0.00235567	0.00061069	0.00512577	0.00347321	0.00255933	0.80372579	0.13943182

8_0	0.01206346	0.01301928	0.00806208	0.00163553	0.01527412	0.0122962	0.00926565	0.6430741	0.25525941
8_1	0.00391627	0.00395938	-0.0006327	0.00117296	0.0052651	0.00309371	-0.0003236	0.77084798	0.24705658
8_2	0.00439191	0.00517307	0.00457221	0.00103393	0.00460555	0.00535614	0.00472893	0.50091556	0.3652368
8_3	-0.0020119	-0.0014346	-0.0014264	-0.0018628	0.00311436	-0.002249	-0.001053	0.59414661	0.38835958
8_4	0.00914975	0.0091072	0.00302215	0.00335586	0.01228037	0.00924648	0.00291187	0.4714311	0.3559136
9_0	-0.0021164	-0.002177	-0.0003688	-0.0008219	0.00092426	-0.0020905	-0.000735	0.53557386	0.5311423
9_1	0.0087404	0.00773892	0.00560542	0.00307455	0.01265027	0.00899232	0.00527363	0.65655546	0.26229032
9_2	0.02031442	0.02143945	0.00608029	0.00650342	0.0237407	0.01900072	0.0059028	0.57241893	0.44707847
9_3	0.00517609	0.00485198	0.00321994	0.00132536	0.00674452	0.00589615	0.00317543	0.49897216	0.27008889
9_4	0.01250583	0.01171925	0.01204314	-0.0001683	0.01582669	0.01414941	0.01223567	0.62304511	0.30368633
